# Implementing a patient-oriented pole walking intervention in retirement homes: A non-randomized feasibility trial

**DOI:** 10.1371/journal.pone.0349290

**Published:** 2026-06-17

**Authors:** Mohsen Keramati, Mahdi Rostami Haji Abadi, Saija Kontulainen

**Affiliations:** College of Kinesiology, University of Saskatchewan, Saskatoon, Saskatchewan, Canada; UBC: The University of British Columbia, CANADA

## Abstract

**Objectives:**

To evaluate the feasibility and safety of implementing a patient-oriented pole walking (PW) intervention in retirement home settings and preliminary changes in outcome measures related to physical function and other fall- and fracture-related risk factors to inform a future randomized controlled trial (RCT).

**Methods:**

This single-arm, non-randomized feasibility trial implemented a patient-oriented PW intervention across four retirement homes in Saskatoon, Saskatchewan, Canada. During Summer 2022, we assessed 24 residents for eligibility, of which 19 consented and 17 received the intervention. The intervention was offered as supervised group sessions (20–60 minutes) three times per week for 12 weeks. Each session consisted of posture and balance warm-up, PW, muscle strengthening, and stretching. The primary outcome measure was feasibility as assessed by consent, recruitment, retention, and adherence rates as well as by intervention acceptability, appropriateness, and feasibility scores. The secondary outcome measures included safety (evaluated by recorded adverse events) and preliminary 12-week changes in physical function and other fall- and fracture-related risk factors (examined with paired-samples t-tests or repeated measures analysis of covariance models).

**Results:**

Fifteen participants (mean age 85.2 years; 93% female) completed the study. The consent, recruitment, retention, and mean adherence rates were 79%, 2.7 participants/site/month, 88%, and 90%, respectively. The mean participant- and instructor-reported scores for intervention acceptability, appropriateness, and feasibility were all > 4.0 (out of 5). There were no recorded intervention-related serious adverse events. Participants improved their functional balance/mobility (timed “up & go” test: −1.6 seconds; 95% CI: −2.7 to −0.4), lower-body strength (30-second chair stand test: 2.4 repetitions; 1.2 to 3.5), 36-item short-form survey physical functioning score (12.9; 3.7 to 22.2), and forearm muscle area (67.7 mm^2^; 12.9 to 122.6) over 12 weeks.

**Conclusions:**

It was feasible and safe to implement our patient-oriented PW intervention in retirement homes. Findings will inform our future RCT in these settings.

**Trial registration:**

ClinicalTrials.gov NCT05388227.

## Introduction

The global burden of falls and related fractures in the older population is significant [1,2]. Compared with community-dwelling older adults, those in residential care settings (hospitals, long-term care facilities, and retirement homes) often face more complex health challenges that put them at up to twice the risk of falling [3–6]. Systematic reviews of randomized controlled trials (RCTs) consistently show that exercise interventions, particularly when targeting balance, gait, and muscle strength, reduce the rate of falls and the number of those who fall or sustain fall-related fractures among older individuals living in the community [7–9]. However, evidence on the efficacy of such interventions in residential care settings has been inconclusive and sometimes controversial, leading to a call for further exercise RCTs [9–12].

A particular form of exercise that has become increasingly attractive to older adults in recent years is Nordic/pole walking (PW), a moderate-intensity aerobic activity in which the active use of handheld poles while walking increases upper-body engagement [13–15]. To our knowledge, no RCT has directly assessed the efficacy of PW interventions in preventing falls or fractures. However, a systematic review by Bullo et al. [16] suggests that these interventions may enhance functional balance/mobility and muscle strength in community-dwelling older adults—physical function measures known to protect against falls and related fractures [3,4,17–19]. Within residential care settings, the evidence remains limited to only two RCTs which examined the efficacy of PW as a standalone intervention on modifiable risk factors for falls- and fall-related fractures. Fritschi et al. [20] (12 weeks PW; three 20-minute sessions per week) reported no changes in physical function or health-related quality of life (HRQoL), whereas Nawrat-Szołtysik et al. [21,22] (12 months PW; two 30–45-minute sessions per week) observed improvements in both. Given these inconsistent findings and the overall paucity of evidence, further RCTs are needed to establish the efficacy of PW interventions in preventing falls and related fractures or reducing their modifiable risk factors among older adults in residential care.

Retirement homes are privately paid residences for older individuals who can generally manage their own care but may require assistance with some activities of daily living, often providing different levels of care from independent living to more supportive units [6,23]. They constitute an important and substantial transitional stage within the residential care continuum, especially in Canada where nearly as many older adults reside in retirement homes as in hospitals and long-term care facilities combined [6,24]. Nevertheless, most fall-prevention research to date has focused on long-term care facilities, leaving retirement homes largely understudied [6]. Thus, we conducted this study to evaluate the feasibility of implementing a patient-oriented PW intervention in retirement home settings as the primary outcome, and to assess safety and preliminary changes in physical function and other fall- and fracture-related risk factors as secondary outcomes, to inform a future RCT.

## Materials and methods

### Design

This was a multi‑site, single‑arm, non‑randomized feasibility trial implementing a patient‑oriented PW intervention. We prospectively registered the study protocol (S1 File) on ClinicalTrials.gov on May 24, 2022 (ID: NCT05388227; https://clinicaltrials.gov/study/NCT05388227), and the University of Saskatchewan (USask) Biomedical Research Ethics Board approved it on 26 May 2022 (ID: 3414). All participants provided written informed consent before inclusion. As retirement home residents were deemed capable of making independent decisions, we did not perform a formal assessment of capacity to consent, in accordance with the Ethics Board approval. The study was conducted according to the principles expressed in the Declaration of Helsinki. Reporting followed the CONSORT 2010 extension to randomized pilot and feasibility trials [25], adapted for a non-randomized design [26], as well as the TREND Statement [27] and TIDieR Guide [28]. The TREND (S2 File) and TIDieR Checklists (S3 File) are provided as supporting information.

### Setting and participants

**Fig 1:**
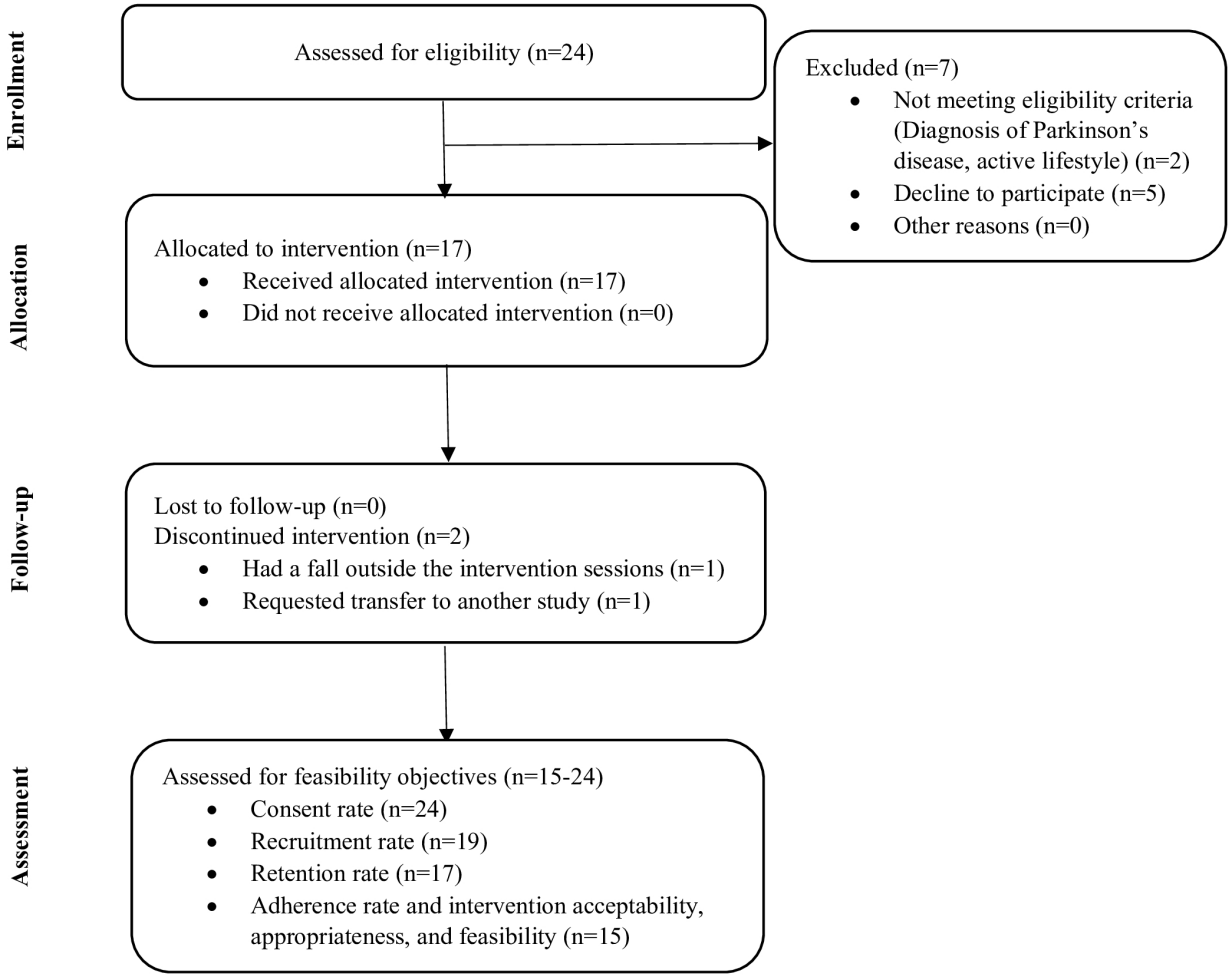
Participant flow throughout the study.

Between June 28 and August 19, 2022, we identified 24 potentially eligible residents (Fig 1) from four retirement homes in Saskatoon, Saskatchewan, Canada, through study posters and flyers placed in each site’s public areas, followed by one group presentation at each site. We identified retirement homes from publicly available listings and prior community contacts and approached them by email. Sites and participants were recruited using a convenience sampling approach. A total of 24 residents received the written informed consent forms, of whom 19 (n = 5 in sites 1 and 2, n = 2 in site 3, and n = 7 in site 4) subsequently provided consent and were recruited (mean age 83.8 years; 84% female). To instruct the intervention sessions, we recruited 14 student volunteers from the USask College of Kinesiology via email, without a predefined recruitment target. According to the Ethics Board approval, student volunteers were not required to provide written informed consent.

A trained research assistant (RA) performed eligibility screening, obtained written informed consent, and consecutively enrolled eligible participants. Inclusion criteria were being an ambulatory retirement home resident and passing the Get Active Questionnaire [29]. This questionnaire is a self-report tool commonly used in Canada to determine whether individuals can safely engage in physical activity [29]. To pass, participants had to answer “No” to all four questions, and if they answered “Yes” to any, they were required to provide Physician Physical Activity Readiness Clearance forms signed by their family physician before inclusion. Exclusion criteria included using assistive devices for mobility, being active (moderate-to-vigorous physical activity ≥150 minutes per week based on accelerometry), and a diagnosis of Parkinson’s disease.

All baseline and follow-up data collection sessions took place at our lab in the USask College of Kinesiology, with the final follow-up session occurring on November 24, 2022. Transportation to and from the lab was provided to all participants. All questionnaires used for data collection were paper-based.

### Intervention

In addition to student volunteers, 1–2 participants from each site volunteered to lead the intervention sessions as peer instructors. All student- and peer instructors received training before the intervention began, led by the principal investigator (S.K.) and/or a trained RA, as planned in the study protocol (S1 File). Each instructor attended a single 1-hour training session. In addition, during the first week of the intervention, instructors were accompanied by a trained RA to ensure that sessions were delivered as intended. The intervention consisted of supervised group sessions offered at participating sites three times per week for 20–60 minutes per session over 12 weeks, held outdoors when possible and indoors if weather did not permit. The intervention was implemented between July and November 2022, with staggered initiation across sites. All sites had safe and accessible outdoor walking paths, as well as indoor walking areas when needed. At least one trained instructor was present at every session. Participants and instructors used study-provided walking poles (professional edition ACTIVATOR poles, Urban Poling Inc., Vancouver, BC, Canada). Poles were provided at each site at the start of the intervention for shared use during the intervention period only and were not assigned to individuals. We instructed participants to use the poles only during supervised sessions, although use outside these sessions could not be verified. Other physical activity outside the intervention sessions was not formally monitored to minimize participant burden.

**Fig 2:**
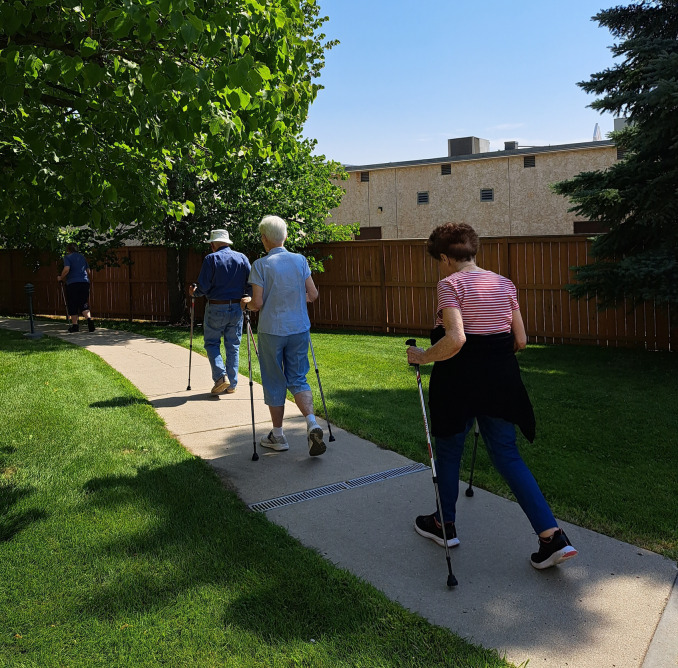
Pole walking.

Table 1 presents the overall structure of the intervention sessions, implemented based on the patient‑oriented USask Nordic Walking Study [30], an RCT co‑designed with patient-partners with lived experience of osteoporosis, prior vertebral fracture, or hyperkyphosis, as well as health care providers and decision-makers. In line with the recommendations on exercise for fall and fracture prevention for older adults [31], every session included multiple components performed with poles in the following order: posture and balance warm-up, PW (Fig 2), muscle strengthening, and stretching. The PW and muscle strengthening exercises were progressive with staged increases and flexibility based on participant tolerance. We provided each participant and instructor with a visual exercise guide outlining session structure, safe exercise techniques, and a link to a video of an example session (https://www.youtube.com/watch?v=fJCIr6q8Dlw). Instructors recorded attendance in individual logbooks dedicated to each participant.

**Table 1:** Overall structure of the implemented intervention sessions^a, b^.

**Posture and balance warm-up**	Week 1–12: 3-5 min • Posture with poles (~30 s) • Shoulder rolls (10 reps, forward and backward) • Toe-to-heel balance (~30 s) • Single-leg balance (~30 s, each leg)			
**Pole walking** ^**c**^	Week 1–3: 15-20 min	Week 4–6: 20-30 min	Week 7–9: 30-35 min	Week 10–12: 35-45 min
**Muscle strengthening exercises** ^**c**^	Week 1–3: 3-5 min • Squats (5–8 reps) • Lunges (5–8 reps, each leg)	Week 4–6: 3-5 min • Squats (9–12 reps) • Lunges (9–12 reps, each leg)	Week 7–9: 3-5 min • Squats (7–9 reps) • Lunges (7–9 reps, each leg) • Rest • Squats (7–9 reps) • Lunges (7–9 reps, each leg)	Week 10–12: 3-5 min • Squats (10–12 reps) • Lunges (10–12 reps, each leg) • Rest • Squats (10–12 reps) • Lunges (10–12 reps, each leg)
**Stretching exercises**	Week 1–12: 3-5 min • Calf stretch (~30 s, each leg) • Hip flexors stretch (~30 s, each leg) • Hamstring and lower back stretch (~30 s) • Chest stretch (~30 s)			

^a^ The University of Saskatchewan Nordic Walking Study Exercise Guides: https://research-groups.usask.ca/nws/nordic_walking_study.php#NordicWalkingExerciseGuides.

^b^ Participants used walking poles for all components of the intervention.

^c^ Pole walking and muscle strengthening exercises were progressive with staged increases and flexibility based on participant tolerance.

### Participant characteristics

We collected data on age (years), sex (female or male), and fall history in the past 12 months (yes or no) using a general self-report questionnaire. Falls were defined as any event where any part of the body unexpectedly contacted the ground or another lower surface [32].

A single trained RA performed all anthropometric measurements in accordance with the standards for anthropometric assessment [33]. Height was measured with a wall-mounted stadiometer (Harpenden 602VR, Holtain Ltd., Crymych, Dyfed, UK) and weight with a mechanical scale (Toledo Scale Company Ltd., Windsor, ON, Canada). We calculated BMI as weight in kilograms divided by height in meters squared. Radius and tibia lengths of the non-dominant limbs were measured with a flexible segmometer (Model 4, Rosscraft Innovations Inc., Surrey, BC, Canada).

A certified medical radiation technologist acquired and analyzed dual-energy X-ray absorptiometry (DXA) scans of participants’ left hip and lumbar spine at baseline (QDR Series, Discovery W, Hologic Inc., Bedford, MA, USA). Osteoporosis was defined as a T-score of ≤–2.5 at the left femoral neck or lumbar spine [34].

### Feasibility outcome measures

The primary outcome measure was feasibility as assessed by consent, recruitment, retention, and adherence rates as well as by participant- and instructor-reported intervention acceptability, appropriateness, and feasibility scores. Consent rate was defined as the percentage of participants assessed for eligibility who provided consent [35,36]. We calculated recruitment rate (participants/site/month) by dividing the number of consenting participants by the number of recruiting sites and the total recruitment period in months [35,36]. Retention rate was defined as the percentage of enrolled eligible participants who received the intervention and completed both baseline and follow-up data collection sessions [35,36]. We calculated adherence rate as the percentage of available intervention sessions each participant attended [37].

At follow-up data collection sessions, participants rated the intervention’s acceptability, appropriateness, and feasibility using three valid and reliable self-report scales developed by Weiner et al. [38]. Each 4-item scale provides a score from 1 to 5, with higher scores indicating greater acceptability, appropriateness, or feasibility of an intervention [38]. The same scales were also emailed as a single file (feasibility survey) to all 14 student instructors after the last intervention session. In accordance with the Ethics Board approval, the survey included an information statement indicating that participation was voluntary, responses would be anonymized, and that responses would be used in the evaluation of the intervention.

### Secondary outcome measures

The secondary outcome measures included safety and preliminary changes in the following fall- and fracture-related risk factors (assessed at both baseline and follow-up): physical function (functional balance/mobility, lower- and upper-body strength, functional capacity), HRQoL, physical activity and sedentary time, fear of falling, and musculoskeletal parameters derived from peripheral quantitative computed tomography (pQCT).

To evaluate safety, a trained RA completed an adverse event form for each participant at their follow-up session (active monitoring), recording every event that occurred during or after the intervention, along with its seriousness and relatedness to the intervention. Adverse event forms were not completed at each intervention session. However, if an adverse event occurred at any time during the intervention period, it was reported by instructors, and the RA promptly contacted the participant (in person or by phone) to complete the form (spontaneous monitoring). Adverse events were defined as unfavorable or harmful outcomes that occur during or after an intervention but are not necessarily caused by it [39,40]. Serious events were those resulting in death, being life-threatening, requiring hospitalization, or causing persistent or significant disability [40]. Intervention-related events were those deemed possibly, probably, or definitely related to the intervention by the affected participants [40].

We asked participants to perform the timed “up & go” test (TUG) three times with short rest intervals. This test is a valid and reliable tool for evaluating functional balance and mobility in older adults, in which participants are timed as they rise from a standard armchair, walk three meters, turn, walk back, and sit down at their usual pace [41]. We reported the best TUG time in seconds.

In addition, participants completed the 30-second chair stand test (30CST), a valid and reliable measure of lower-body strength in the older population [42]. Sitting on a standard armless chair, they were instructed to rise to a full stand without using their arms and sit down again as many times as possible within 30 seconds [42]. We recorded the number of correctly executed full stands.

Furthermore, participants performed the hand grip strength test, a valid and reliable measure of upper-body strength among older adults [43,44], using a JAMAR Hydraulic Hand Dynamometer (Patterson Medical, Warrenville, IL, USA). While seated on a standard armchair, they were asked to squeeze the dynamometer as hard as possible for three seconds, three times with each hand, with at least 30 seconds of rest between attempts [44]. The best results for each hand were averaged together and reported to the nearest kilogram.

Moreover, we instructed participants to complete the 6-minute walk test, which is a valid and reliable tool for assessing functional capacity [45] and a quick, safe indicator of physical fitness in older individuals [46]. In this test, the distance a participant can walk on a flat, hard surface in six minutes is recorded in meters [45]. Participants could choose their walking pace and were allowed to stop and rest if needed [45]. A trained RA conducted all physical function tests in this study.

Each participant completed the 36-item short-form survey (SF-36; RAND Corporation, Santa Monica, CA, USA), a valid and reliable self-report questionnaire for assessing HRQoL in older adults [47]. The SF-36 yields a score from 0 to 100 for each of its eight scales, with higher scores indicating more favorable health states [48].

We measured time spent per day in sedentary, light (LPA), and moderate-to-vigorous (MVPA) physical activity with triaxial accelerometers (ActiGraph wGT3X-BT, ActiGraph LLC, Pensacola, FL, USA) [49,50]. Participants were instructed to wear the accelerometer for 7 consecutive days during waking hours, remove it during water-based activities (e.g., bathing or swimming), and complete a daily wear time log indicating periods when the device was removed to support wear time validation [49,50]. Non-wear time was defined as ≥20 minutes of consecutive zero accelerometer counts, and the minimum wear time per day was set at 480 minutes [49,50]. We included participants with at least two weekdays and one weekend day of valid data in the accelerometry analysis [49,50]. MVPA, LPA, and sedentary time were classified according to cutoffs of ≥1952, 100–1951, and <100 counts per minute, respectively [49–51].

Participants completed the 10-item falls efficacy scale, which is a valid and reliable self-report measure of fear of falling among older adults [52]. The FES provides a total score ranging from 10 to 100, with higher scores indicating greater fear of falling [52].

A trained RA obtained and analyzed pQCT scans (XCT 2000, STRATEC Medizintechnik GmbH, Pforzheim, Germany) of each participant’s non-dominant forearm and lower leg according to our standard protocols [53,54]. This imaging modality provides three-dimensional measures of bone density, geometry, and estimated strength, separately for trabecular and cortical compartments, as well as muscle area and density at the peripheral skeleton, thereby enabling assessment of musculoskeletal health beyond areal bone density measures obtained by DXA [53–55]. Distal sites were scanned at 4% of the radius and tibia length, and shaft sites were scanned at 65% of the radius length and 66% of the tibia length [53,54]. For distal scans, we reported total and trabecular area (mm^2^), total and trabecular density (mg/cm^3^), and estimated bone strength in compression (mg^2^/mm^4^) [53–55]. For shaft scans, we reported total and cortical area, cortical density, estimated bone strength in torsion (mm^3^), and forearm/lower leg muscle area and density [53–55].

### Sample size

As this was a feasibility study, we did not conduct a formal power calculation [56,57]. Guidelines for designing and evaluating feasibility studies state that sample sizes should be adequate to reasonably address feasibility objectives [56–58]. The literature recommends 12–35 participants per intervention group to achieve this goal [58,59]. Our final sample size of 15 participants falls within this recommended range.

### Statistical analysis

Feasibility and safety outcomes were reported descriptively and/or narratively. As this was a feasibility study, no formal hypothesis testing was planned. However, for exploratory purposes, we used paired-samples t-tests to examine preliminary 12-week changes in physical function, HRQoL, fear of falling, and pQCT-derived parameters. Changes in physical activity and sedentary time were evaluated using repeated-measures analysis of covariance models, with the difference in wear time between baseline and follow-up included as a covariate. We did not account for the clustered nature of the data (participants nested within sites) in these analyses, as the study was not powered for such analyses and was only intended to inform a future cluster-RCT. All statistical analyses were performed in SPSS Statistics, version 27 (IBM Corporation, Armonk, NY, USA).

### Patient and public involvement

This study implemented the USask Nordic Walking Intervention [30]. The intervention was originally co-designed with patient-partners with lived experience of osteoporosis, prior vertebral fracture, or hyperkyphosis, as well as health care providers and decision-makers, in alignment with the Canadian Institutes of Health Research Strategy for Patient-Oriented Research (SPOR) principles [30,60]. Patient partnership in the original intervention focused on ensuring relevance, safety, acceptability, and feasibility for older adults at increased risk of fractures [30].

The present feasibility study evaluated the implementation of this patient‑oriented intervention in retirement homes. The study was initiated following requests from residents of several retirement homes who expressed interest in having a Nordic/pole walking program offered within their facilities, reflecting patient‑identified priorities at the implementation level. In addition, 1–2 participants at each site volunteered to be trained as peer instructors and actively contributed to delivering the intervention by leading group PW sessions. While these participants were not involved in the study design, governance, or analysis, their peer‑led involvement supported program delivery and engagement. Together, this approach was consistent with SPOR guidance by building on a patient‑partner‑designed intervention and responding to priorities identified by end users during implementation, with the overarching goal of improving outcomes relevant to older adults at elevated fracture risk [60].

## Results

### Participant flow throughout the study

We excluded one participant due to a diagnosis of Parkinson’s disease and another because of an active lifestyle, leaving 17 eligible participants (mean age 84.2 years; 88% female) to receive the intervention (Fig 1). Of these, two discontinued—one female after a fall outside the intervention sessions and one male who requested transfer to another study—leaving 15 (mean age 85.2 years; 93% female) who completed both baseline and follow-up data collection sessions (Fig 1). The completers had a mean body mass index (BMI) of 25.7 kg/m^2^, 60% were osteoporotic, and 47% had fallen in the past 12 months.

### Feasibility

The consent, recruitment, retention, and mean adherence rates were 79%, 2.7 participants/site/month, 88%, and 90%, respectively (Table 2). The mean participant-reported scores for intervention acceptability, appropriateness, and feasibility were 4.3, 4.0, and 4.1 (out of 5), respectively (Table 2). Eight out of 14 student instructors responded to the emailed feasibility survey, with mean scores of 4.9 (out of 5) for all three scales (Table 2).

**Table 2:** Feasibility results of the study^a^.

Outcome measure	Finding
**Consent rate (%)**	79.2
**Recruitment rate (participants/site/month)**	2.7
**Retention rate (%)**	88.2
**Adherence rate (%), mean (SD)**	90.2 (11.0)
**Participant-reported scores (out of 5)**	
**Intervention acceptability, mean (SD)**	4.3 (1.0)
**Intervention appropriateness, mean (SD)**	4.0 (0.9)
**Intervention feasibility, mean (SD)**	4.1 (1.0)
**Instructor-reported scores**^**b**^ **(out of 5)**	
**Intervention acceptability, mean (SD)**	4.9 (0.3)
**Intervention appropriateness, mean (SD)**	4.9 (0.2)
**Intervention feasibility, mean (SD)**	4.9 (0.3)

^a^ n = 24 for consent rate; n = 19 for recruitment rate; n = 17 for retention rate; n = 15 for adherence rate and participant-reported scores; n = 8 for instructor-reported scores.

^b^ Instructor-reported scores represent student instructors only, as peer instructors were also participants.

### Safety

Seven participants experienced adverse events. Of these, six reported non-serious events such as musculoskeletal pain in the hips, shoulders, back, and ankles, while one had a fall outside the intervention sessions that required hospitalization (a non-intervention-related serious event). Five participants believed that their events were related to the intervention. There were no recorded intervention-related serious adverse events.

### Preliminary changes in fall- and fracture-related risk factors

Participants improved their functional balance/mobility (TUG: −1.6 seconds; 95% CI: −2.7 to −0.4), lower-body strength (30CST: 2.4 repetitions; 1.2 to 3.5), SF-36 physical functioning score (12.9; 3.7 to 22.2), and forearm muscle area (67.7 mm^2^; 12.9 to 122.6) over 12 weeks (Table 3). We also observed reductions in SF-36 general health score (−7.4; −14.4 to −0.4) and tibia shaft cortical area (−6.4 mm^2^; −11.2 to −1.6) (Table 3). No changes were observed in physical activity and sedentary time, fear of falling, other physical function measures, remaining SF-36 scale scores, or other pQCT-derived parameters over the 12-week intervention period (Table 3).

**Table 3:** Pre- and post-intervention results for physical function, HRQoL, physical activity and sedentary time, fear of falling, and pQCT-derived parameters^a,b,c^.

	Variable	Pre-intervention, mean (SD)	Post-intervention, Mean (SD)	Pre-post change Mean (95% CI)
**Physical function**	TUG (s)	13.8 (6.1)	12.2 (5.1)	−1.6 (−2.7; −0.4)
	30CST (reps)	10.0 (2.9)	12.4 (3.0)	2.4 (1.2; 3.5)
	HGS (kg)	15.4 (5.3)	16.5 (5.8)	1.1 (−0.5; 2.8)
	6MWT (m)	287.6 (118.7)	284.5 (112.2)	−3.1 (−38.5; 32.3)
**HRQoL**	SF-36 physical functioning score	54.1 (27.0)	67.0 (21.7)	12.9 (3.7; 22.2)
	SF-36 role functioning/physical score	55.5 (38.8)	56.7 (38.3)	1.1 (−21.0; 23.2)
	SF-36 role functioning/emotional score	66.7 (39.9)	66.7 (39.8)	0.0 (−22.0; 22.0))
	SF-36 energy/fatigue score	54.0 (20.6)	59.0 (22.1)	5.0 (−4.5; 14.5)
	SF-36 emotional well-being score	68.5 (22.1)	70.9 (24.2)	2.4 (−3.4; 8.2)
	SF-36 social functioning score	71.7 (24.3)	76.7 (24.9)	5.0 (−9.6; 19.5)
	SF-36 pain score	68.5 (23.0)	63.4 (20.4)	−5.2 (−15.3; 5.0)
	SF-36 general health score	65.7 (23.1)	58.3 (21.5)	−7.4 (−14.4; −0.4)
**Physical activity and sedentary time**	LPA (min/day)	112.5 (29.2)	111.5 (48.7)	−1.0 (−19.1; 17.1)
	MVPA (min/day)	6.8 (4.7)	8.8 (8.7)	2.0 (−3.7; 7.7)
	Sedentary time (min/day)	727.0 (139.0)	639.8 (73.7)	−87.2 (−104.8; −69.6)
**Fear of falling**	FES score	24.0 (15.5)	27.7 (19.4)	3.7 (−2.7; 10.1)
**pQCT-derived parameters**				
**Distal radius**	Total area (mm^2^)	430.5 (59.4)	436.2 (53.7)	5.6 (−9.4; 20.7)
	Total density (mg/cm^3^)	184.6 (38.0)	181.9 (35.9)	−2.7 (−6.8; 1.4)
	Trabecular area (mm^2^)	423.7 (63.1)	429.9 (56.0)	6.2 (−10.0; 22.4)
	Trabecular density (mg/cm^3^)	177.4 (33.6)	175.3 (32.8)	−2.1 (−4.5; 0.2)
	Estimated bone strength in compression (mg^2^/mm^4^)	15.1 (5.2)	15.0 (5.1)	−0.1 (−0.5; 0.2)
**Distal tibia**	Total area (mm^2^)	1175.7 (150.5)	1157.0 (155.6)	−18.7 (−40.0; 2.6)
	Total density (mg/cm^3^)	191.7 (38.6)	193.1 (37.2)	1.4 (−2.8; 5.6)
	Trabecular area (mm^2^)	1153.2 (143.5)	1133.3 (144.9)	−19.9 (−44.1; 4.3)
	Trabecular density (mg/cm^3^)	184.0 (38.7)	185.0 (38.2)	0.9 (−2.7; 4.6)
	Estimated bone strength in compression (mg^2^/mm^4^)	44.7 (17.8)	44.8 (17.6)	0.0 (−1.5; 1.6)
**Radius shaft**	Total area (mm^2^)	129.6 (17.9)	131.2 (18.0)	1.6 (−2.5; 5.7)
	Cortical area (mm^2^)	74.5 (16.4)	75.1 (18.9)	0.6 (−2.8; 4.1)
	Cortical density (mg/cm^3^)	976.1 (71.7)	971.3 (70.2)	−4.8 (−42.7; 33.0)
	Estimated bone strength in torsion (mm^3^)	231.7 (65.4)	230.7 (63.4)	−1.0 (−8.8; 6.7)
**Tibia shaft**	Total area (mm^2^)	579.8 (83.6)	579.7 (81.9)	−0.1 (−22.4; 22.2)
	Cortical area (mm^2^)	261.9 (52.0)	255.4 (51.3)	−6.4 (−11.2; −1.6)
	Cortical density (mg/cm^3^)	997.4 (45.4)	1004.7 (51.1)	7.2 (−12.7; 27.1)
	Estimated bone strength in torsion (mm^3^)	1885.9 (333.9)	1862.9 (327.3)	−23.0 (−57.5; 11.5)
**Forearm**	Muscle area (mm^2^)	2342.4 (663.9)	2410.2 (703.6)	67.7 (12.9; 122.6)
	Muscle density (mg/cm^3^)	72.9 (3.7)	73.7 (4.2)	0.8 (−2.0; 3.5)
**Lower leg**	Muscle area (mm^2^)	5035.4 (830.0)	5013.2 (720.7)	−22.2 (−223.3; 178.8)
	Muscle density (mg/cm^3^)	66.1 (3.3)	65.0 (3.3)	−1.1 (−2.4; 0.2)

HRQoL, health-related quality of life; pQCT, peripheral quantitative computed tomography; TUG, timed “up & go” test; 30CST, 30-second chair stand test; HGS, hand grip strength test; 6MWT, 6-minute walk test; SF-36, 36-item short-form survey; LPA and MVPA, light and moderate-to-vigorous physical activity; FES, falls efficacy scale.

^a^ n = 15 for TUG, HGS, HRQoL, and fear of falling; n = 14 for 30CST and 6WMT; n = 8 for physical activity/sedentary time; n = 11 for distal radius, radius shaft, and forearm; n = 12 for distal tibia; n = 10 for tibia shaft and lower leg.

^b^ Values are calculated using paired-samples t-tests for all variables except physical activity and sedentary time.

^c^ For physical activity and sedentary time, values are calculated using repeated measures analysis of covariance models with the difference in wear time between baseline and follow-up included as a covariate.

## Discussion

Our primary objective was to evaluate the feasibility of implementing a patient-oriented PW intervention in retirement home settings. The secondary objectives were to assess safety and explore preliminary changes in outcome measures related to physical function and other fall- and fracture-related risk factors to inform a future RCT. In terms of implementation feasibility, we observed consent, recruitment, retention, and mean adherence rates of 79%, 2.7 participants/site/month, 88%, and 90%, respectively. In addition, participants and instructors rated the intervention highly (mean scores >4.0 out of 5) for acceptability, appropriateness, and feasibility. Furthermore, no serious intervention-related adverse event occurred, supporting its safety. We also observed preliminary improvements in functional balance/mobility, lower-body strength, SF-36 physical functioning score, and forearm muscle area, while tibia shaft cortical area and SF-36 general health score declined, informing our future RCT.

Our consent rate of 79% was higher than the median values reported in systematic reviews of RCTs by Jacques et al. (72%) [35] and Walters et al. (70%) [36]. Similarly, the recruitment rate of 2.7 participants/site/month was higher than the medians reported in these reviews (1.0 and 0.9 participants/site/month, respectively) [35,36]. In addition, the retention rate of 88% was comparable to those of the two RCTs of PW interventions in residential care settings by Fritschi et al. (73%) [20] and Nawrat-Szołtysik et al. (98%) [21,22], and also fell within the 77–94% range reported by Weber et al.’s review [61] for walking-based exercise programs within residential care settings as well as the ≥ 80% threshold considered acceptable for clinical trials in evidence-based medicine [62,63]. Likewise, our mean adherence rate of 90% was comparable to the 78–80% range reported by Weber et al. [61] and exceeded the minimum acceptable adherence threshold of 70% recommended by the National Center for Complementary and Integrative Health [58]. Finally, mean participant- and instructor-reported scores of >4.0 (out of 5) for intervention acceptability, appropriateness, and feasibility indicated that, on average, respondents either agreed or completely agreed that the intervention was acceptable, appropriate, and feasible [38]. Collectively, the feasibility findings of this study demonstrated that it was feasible to implement our patient-oriented PW intervention in retirement home settings.

Our feasibility results also provide essential guidance for the design and conduct of the future RCT. Considering the consent and recruitment rates, we will likely need to approach a greater number of retirement homes and identify more potentially eligible residents at each site to achieve the required sample size within an efficient time frame. Strengthening recruitment strategies—for example, by engaging staff and resident representatives from participating sites—may further enhance recruitment and consent rates. Furthermore, our observed retention rate will be incorporated into the sample size calculation to account for anticipated dropout and preserve statistical power. In contrast, the excellent adherence rate and consistently high participant- and instructor-reported scores for intervention acceptability, appropriateness, and feasibility suggest that no modifications to the intervention protocol are needed for our future RCT.

Non-serious musculoskeletal pain was the only intervention-related adverse event recorded in this study. This finding aligns with Fritschi et al.’s RCT [20], which also reported non-serious musculoskeletal pain as the only adverse event related to PW among residential care residents, and with evidence from systematic reviews of RCTs suggesting that PW is a safe exercise option for older adults [16,64]. Together, these results indicate that our intervention protocol can be safely implemented in the future RCT.

Due to the study’s design, the observed preliminary improvements in functional balance/mobility, lower-body strength, and SF-36 physical functioning score cannot be attributed to the PW intervention and require testing in our future RCT. Nevertheless, these findings are consistent with Bullo et al.’s systematic review [16], which indicates that PW interventions may improve all three measures in community-dwelling older adults, as well as Nawrat-Szołtysik et al.’s RCT [21,22], which reported similar improvements in functional balance/mobility and HRQoL among residential care residents following PW.

The TUG and 30CST are among the physical function measures widely recommended for fall risk assessment in older adults [65,66], while the SF-36 captures a meaningful patient-reported outcome (HRQoL) that is particularly important for individuals in residential care settings [67]. In addition, the improvements observed in the TUG, 30CST, and SF-36 physical functioning score suggest that these measures may be sensitive to change over a 12-week period. Taken together, we believe it is reasonable to consider changes in these measures as the primary outcomes for the future RCT. Accordingly, because our future RCT will follow a cluster-randomized design, the intracluster correlation coefficients estimated from the observed preliminary changes in these measures, together with information on cluster size and its variability (the average number of eligible participants enrolled per site and the corresponding coefficient of variation), will be used to estimate the design effects required for sample size calculation.

Despite preliminary improvements in these important physical function measures, participation in our structured exercise program did not translate into changes in accelerometer-derived free-living physical activity or sedentary time outside of the intervention period. This likely reflects documented factors beyond physical function that shape activity behavior in older adults in residential care, including contextual influences beyond the individual, such as social and environmental factors within these settings [68]. For the future RCT, addressing these factors, along with additional behavioral change strategies, may help increase free-living physical activity and reduce sedentary time in retirement home residents.

Although we observed an improvement in the SF-36 physical functioning score, there was a reduction in the SF-36 general health score. This contrasts with evidence from PW interventions among older adults [13,15,16,22] and warrants further investigation in the future RCT. Similarly, contrary to evidence in older adults [14,16], we did not find any improvement in upper-body strength. However, we observed an increase in pQCT-derived forearm muscle area, a significant predictor of upper-body strength among older adults [69,70]. Our future RCT will determine whether this preliminary finding can be replicated and whether it is attributable to the PW intervention.

We noted a decrease in cortical area at the tibia shaft. This preliminary finding may reflect trabecularization of the cortex—an age-related process, more pronounced in females than in males, in which endocortical resorption leaves cortical remnants resembling trabecular bone [71,72]. This process typically leads to greater cortical area loss at the weight-bearing tibia than at the radius [70–72]. Moreover, longitudinal pQCT data in older adults indicate that changes in cortical area at the radius and tibia are directly associated with changes in muscle area of the corresponding limb, emphasizing the muscle–bone relationship [70]. Overall, these observations may help explain the sustained cortical area at the radius shaft, possibly due to the observed increase in forearm muscle area. However, these preliminary observations require testing in future RCTs.

The main strength of this multi-site feasibility trial was the implementation of a patient-oriented exercise intervention originally co-designed to address patient-identified priorities related to fracture risk [30]. This patient-oriented foundation enhanced relevance for end users, and engagement during implementation was further supported by empowering participants to serve as peer instructors. However, the study’s single-arm, non-randomized design limits causal inference, as the observed 12-week changes in physical function and other fall- and fracture-related risk factors cannot be attributed solely to the PW intervention and should be considered preliminary and interpreted with caution [73].

## Conclusions

In conclusion, this feasibility study showed high consent, recruitment, retention, and adherence rates along with strong participant and instructor ratings for the acceptability, appropriateness, and feasibility of our patient-oriented PW intervention, demonstrating its implementation feasibility in retirement home settings. No serious intervention-related adverse event occurred, indicating its safety. Preliminary results suggest potential improvements in physical function and forearm muscle area as well as declines in tibia shaft cortical area and SF-36 general health score, highlighting the need for further testing in future RCTs. Overall, these findings support the viability of and will inform the design and conduct of a future RCT to evaluate the efficacy and safety of our PW intervention in improving physical function and other fall- and fracture-related risk factors among retirement home residents.

## Supporting information

S1 File Study protocol. (PDF)

S2 File TREND Checklist. (PDF)

S3 File TIDieR Checklist. (PDF)
