# Supplementary material for: Implementing a patient-oriented pole walking intervention in retirement homes: A non-randomized feasibility trial
Source: PLoS One. 2026 Jun 17;21(6):e0349290. doi: 10.1371/journal.pone.0349290 (PMC13274888; doi:10.1371/journal.pone.0349290)
Supplement: S1 File — (PDF) [file pone.0349290.s001.pdf]

Biomedical Application
Prospective

For Internal Use Only
UnivRS Internal ID:
**Date Received:** Click here to enter a date.

Questions marked with an * are mandatory.

PART 1: KEY INFORMATION

Protocol Number:

Project Title: * **Pole Walking Intervention within Independent Living/Retirement Communities: Feasibility and Piolt**

Level of Risk: * Minimal risk

Expected Start Date: * 2022-06-01

Expected End Date: * 2023-05-31

Explain why this application is time sensitive or specify not applicable (N/A): Time sensitive due to funding cut-off. We need to complete intervention prior to the grant end date.

Applicants

Principal Investigator

Name:	NSID:	Email:	Phone:	Organization (Department):
Saija Kontulainen				College of Kinesiology

Sub-Investigator(s)

Name:	NSID:	Email:	Phone:	Organization (Department):
James (J.D) Johnston				College of Engineering
Kimberly Willison				Saskatchewan Health Authority
Mahdi Rostami				College of Kinesiology

Student(s)

Name:	NSID:	Email:	Phone:	Organization (Department):
Mohsen Keramati				College of Kinesiology

Primary Contact

Name:	NSID:	Email:	Phone:	Organization (Department):
Saija Kontulainen				College of Kinesiology

Secondary Contact

Name:	NSID:	Email:	Phone:	Organization (Department):
**Mahdi Rostami**				**College of Kinesiology**

Sponsor *A sponsor takes responsibility for the initiation and management of research and compliance with applicable regulations. The sponsor may or may not be the main funding organisation.*

Sponsor:
**Saskatchewan Health Research Foundation (SHRF) and Saskatchewan Centre for Patient-Oriented Research (SCPOR)**

Agency(ies) *An agency(ies) provides funding to support research projects. The sponsor may or may not be the main funding organisation.*

This project is funded: * ☒ Yes ☐ No

If yes, the funding supporting this project will be administrated at the University of Saskatchewan: ☒ Yes, complete Part A ☐ No, complete Part B

Part A: For Grants and Contracts administered by the U of S:

Agency:	Pending / Awarded
**Saskatchewan Health Research Foundation (SHRF)**	**Awarded**
**Saskatchewan Centre for Patient-Oriented Research (SCPOR)**	**Awarded**

Project Application(s) Directly Associated with the Fund(s) Supporting this Project.

Please list the UnivRS internal ID# (for pending grants or contracts):

Project(s) Directly Associated with the Fund(s) Supporting this Project

Please list the UnivRS internal ID# (for awarded grants or contracts):

Part B: For Grants or Contracts not administered by the U of S:

Agency:	Pending / Awarded

Pre- and Post-Award Records Directly Linked to this Application

Project application(s) UnivRS internal ID#:
Project(s) UnivRS internal ID #:

Compliance Records Directly Linked to this Application

Compliance Application(s) UnivRS internal ID#:

Describe the relationship between this compliance application and the linked record(s):

Location(s) Where Research Activities Are Conducted

Enter location(s) where this research will be carried out: * **University of Saskatchewan and Independent Living/Retirement Communities in Saskatoon**

Specify country(ies) where the research will be conducted under this Research Ethics Approval: * **Canada**

Other Ethics Approval

Has this project applied for/received approval from any other Research Ethics Board(s) * ☐ Yes ☒ No

If 'yes', identify the other Research Ethics Board(s). For multi-site clinical trials, identify the Canadian sites only or specify 'unknown':

Conflict of Interest

Confirm whether any member of the research team or their immediate family members will:

Receive personal benefits over and above the direct costs of conducting the project, such as remuneration or employment: *	☐ Yes ☒ No
Receive significant payments from the Sponsor such as compensation in the form of equipment, supplies or retainers for ongoing consultation and honorarium: *	☐ Yes ☒ No
Have a non-financial relationship with the Sponsor such as unpaid consultant, board membership, advisor or other non-financial interest: *	☐ Yes ☒ No
Have any direct involvement with the Sponsor such as stock ownership, stock options or board membership: *	☐ Yes ☒ No
Hold patents, trademarks, copyrights, licensing agreements or intellectual property rights linked in any way to this project or the Sponsor: *	☐ Yes ☒ No
Have any other relationship, financial or non-financial, that if not disclosed, could be construed as a conflict of interest: *	☐ Yes ☒ No

If yes was answered to any question(s), explain the personal benefit(s) and how the conflict will be managed:

PART 2: PROJECT OVERVIEW

Brief Overview of Research Project

State the hypothesis or research question(s): * We hypothesize that pole walking intervention within independent living/retirement communities is feasible and will be associated with improved physical activity, physical function, body composition and quality of life and decreased sedentary time in individuals living in independent living/retirement communities.

Summarize in lay terms: a) the background and study rationale; b) the research design; c) methodology; and d) statistical analysis: *

a) the background and study rationale

Loss of muscle (sarcopenia) and bone (osteoporosis) mass and strength in older age increases an individual's risk of falls, fractures and death [1-4]. Falls and fractures significantly contribute to medical care costs worldwide [1-4]. Muscle and bone loss prevention are the cornerstones of fall and fracture prevention in older age [2,5-10]. Hence, exercise is recommended for older people with sarcopenia, osteoporosis, or at increased risk of falling [4,10-13]. Exercise interventions that have improved muscle strength, particularly in the lower extremity[14], have improved balance[15], preventing falls [4,10-13] and fall-related injuries (e.g., fractures)[16,17].

Pole walking provides an attractive form of exercise therapy for older adults. It is a simple, well-tolerated and effective means to improve overall functional fitness in older adults[24,25]. Pole walking improves upper and lower body muscle strength, cardiovascular endurance and flexibility and is recommended as an effective and efficient exercise mode for older adults[24,25]. It also positively affects balance, functional mobility and aerobic exercise capacity[24,25,26]. Moreover, pole walking offers a safe format for walking as poles provide support and help with balance and thus, contribute to confidence in being active. However, there has not yet been a study assessing the feasibility of pole walking intervention in older adults living in independent living/retirement communities.

b) the research design and objectives

This patient-oriented study aims to co-design and pilot a pole walking intervention with residents and staff of the participating independent living/retirement communities. The proposed pilot will answer the following principal question: Is pole walking intervention feasible in the independent living/retirement communities? We will also assess if pole walking intervention will be associated with improved physical activity, physical function, body composition and quality of life and decreased sedentary time in individuals living in independent living/retirement communities.

c) methodology

This study is a patient-oriented pilot intervention. Our target is to include 50 independent living/retirement community's residents. The final format of the intervention will be designed with resident- and staff-advisors.

Pole walking intervention:

Pole walking exercises are based on ongoing, patient-oriented Nordic Walking intervention, tailored for participants and progressive in nature. Training sessions will be led by trained peer/staff/student instructors. Training of instructors will be offered by Dr. Saija Kontulainen (PI, USask) and/or Kimberly Willison (Co-I, SHA), with expertise in Pole/pole walking instructor training. Our research team has developed a video (see link) and exercise guide (attached) detailing exercise protocol and progression for the guidance of participants and to support peer/staff/student instructors leading the sessions.

Group training sessions will be offered at the participating independent living/retirement communities (list attached). Training sessions will be held either indoors or outdoors, 2-3 times a week, for 20-60 min/session, over 12 weeks. We will provide poles to participants/sites. During the first sessions of training with poles, the main goal is to practice a safe walking technique. Each training unit will consist of three phases. During the warm-up phase (5-10 min), participants will perform warm-ups and exercises with poles to improve leg strength, dynamic balance, and posture. The main part of the pole walking training (10-30 min) depends on the participants' skill and comfort level and will consist of walking with poles over a distance to reach an average. The training session will be finished with stretches of the major muscle groups and postural practices (attached exercise guide). Exercises and pole walking will be progressive by increasing the duration of pole walking and the intensity of exercises during the study, with an emphasis on good-quality movement. Participants will be instructed to maintain a Borg Scale intensity of 4–5, based on 70–80% of perceived exertion[30]. Instructors and participants will monitor compliance with the exercise program and other activities using daily activity logs.

Primary outcome measurements:

We will assess feasibility by calculating participant consent and retention rates. In addition, we will assess protocol fidelity (attached survey) by participants and peer/staff/student instructors after the 12-week pole walking intervention at each site.

Secondary outcome measurements:

We will measure participants' muscle strength (sit-to-stand test)[34], grip strength, gait and functional balance (Timed up and go test[32]), walking test, bone and muscle properties and estimated bone strength of the lower leg and forearm (peripheral quantitative computed tomography, pQCT)[35], total body lean and fat tissue mass and bone density at total body, hip and lumbar spine (dual energy X-ray absorptiometry, DXA)[15], exercise confidence [38], fear of falling [39], physical activity (assessed via accelerometers[40]), nutrition (food frequency questionnaire) and quality of life using a validated questionnaire (Short Form [SF-36] questionnaire[32]).

Grip strength: Participants' hand strength will be measured using a special device called a handheld dynamometer. They will be asked to squeeze the device as hard as they can for 3 seconds.

Gait and Functional balance: We will perform 'Timed up and go' test according to the American and British Geriatric Society guidelines as we have reported in our studies with older women[32,33].

Walking test: Participants will complete a 6-minute walking test with and without poles to evaluate exercise capacity in accordance with the American Thoracic Society guidelines. Participants will walk between markers placed 30 m from one other, covering as much distance as possible in 6 minutes. The endpoint for the test is the total distance covered[41].

Imaged bone and muscle properties: Bone and muscle properties and estimated bone strength of the non-dominant forearm and ipsilateral lower leg will be assessed via pQCT (Stratec Medizintechnik GmbH, Pforzheim, Germany) using our standard protocols to quantify muscle cross-sectional area and density[35].

Total body lean and fat tissue mass and areal bone mineral density (aBMD): We will measure lean and fat tissue mass from total body DXA scans and aBMD at total body, hip and lumbar spine to define osteoporosis status according to standard protocols[15].

Exercise confidence: We will assess exercise confidence using the Exercise self-efficacy scale[38]. It uses a 7-point scale (1 - not confident at all to 7- very confident) for 10 different questions about exercises, where a higher score represents a greater confidence.

Fear of falling: This will be measured using the 10-item falls efficacy scale assessing a participant's confidence in accomplishing tasks around the house (e.g., taking a bath or shower)[39]. It uses a 10-point scale (1 - not confident at all to 10- very confident) for 10 different everyday tasks, where a lower score represents a greater fear of falling. This scale is the most effective clinical assessment for fear of falling[4].

Quality of life: We will measure the health-related quality of life using the SF-36 questionnaire as we have previously reported[46].

Tertiary outcome measurements:

We will measure adverse events (e.g., reported fall and injuries) according to the guideline for good clinical practice[55]

Adverse Events: We will probe participants for adverse events at each study visit and fill out adverse event forms[18] that provide details on the type of adverse event, severity, frequency, and relationship to the intervention according to the CONSORT guidelines[42].

Health: We will assess participants' health and medications using a questionnaire.

d) Statistical analysis

We will calculate the retention rate as the percentage of participants who complete the 12-week intervention divided by the total number of participants who consent to the study.

Data from baseline measurements will be compared to those obtained after the 12-week intervention period by the paired sample t-test or Wilcoxon signed-rank test. Alpha will be 0.05. Baseline characteristics of participants that do not adhere to the exercise will be compared to participants that adhere to the program by a one-way analysis of variance to determine if there are any important differences between these groups.

Summarize the anticipated public and scientific benefits of the project: This study will provide new evidence of the feasibility as well as potential benefits of pole walking for the older adults living in independent living/retirement communities. The proposed pilot will provide new and important evidence as it will elucidate the effects of non-pharmacological, exercise intervention on key determinants of fall and fracture risk in the elderly. This information is of great consequence since pharmacological treatments have shown poor efficacy in the prevention of fractures and evidence of non-pharmacological interventions often exclude the elderly with fractures[43]. Benefits for the health care system will be two-fold. First, partnering and empowering patient representatives will likely lead to further collaboration and identification of new patient-originated solutions for healthcare systems and services to facilitate primary and secondary prevention of falls and fractures. Participant-driven, collaborative research processes will lead to improved patient outcomes. Second, we anticipate that beneficial findings from this study will offer a simple, feasible and inexpensive therapy option (walking with poles) that can be broadly implemented into existing and new falls prevention and physical activity programs in the health region. For all elderly, the benefits of this project could be significant. If this pole-walking therapy could lower the risk of falls and fractures by improving mobility, posture, and functional ability, it could improve their quality of life and secure longer independent living at home.

Optional Sub-Studies

Are there any optional sub-studies specified in the protocol? ☐ Yes ☒ No
If yes, describe the purpose and details of each sub-study:

No Objection Letter and/or an Investigational Testing Authorization

Identify whether a No Objection Letter and/or an Investigational Testing Authorization has been received for this project.

Biologics and Genetics Therapies Directorate (BGTD):	☐ Yes ☐ Pending ☒ Not Applicable
Investigational Testing Authorization (ITA):	☐ Yes ☐ Pending ☒ Not Applicable
Natural Health Products Directorate (NHPD):	☐ Yes ☐ Pending ☒ Not Applicable
Therapeutic Products Directorate (TPD):	☐ Yes ☐ Pending ☒ Not Applicable

Other Regulatory Requirements

Is there a requirement for this project to comply with the United States Office for Human Research Protections regulations (OHRP):	☐ Yes ☒ No
Is there a requirement for this project to comply with the United States Food and Drug Administration regulations (FDA):	☐ Yes ☒ No

Is this project being conducted under an Investigational New Drug (IND) application or Investigational Device Exemption (IDE):	☐ Yes ☒ No
Does this project meet the definition of a clinical trial requiring registration?*	☒ Yes ☐ No
If yes, provide the registration number:	NCT05388227
If this project has a Data Safety Monitoring Board, other monitoring systems and/or planned interim analysis, describe, including reporting schedules:	

Procedures and Risks

Identify research-specific procedures that are different from the current standard of care: There is no current standard of care for improving physical activity, physical function, body composition and quality of life or decreasing sedentary time in residents of iliving in the independent living/retirement communities.

Identify any risks associated with research-specific procedures: The minor risks of this study involve muscle pulls or strains, or muscle soreness during or after exercise. There is also a small amount of radiation exposure from the DXA and pQCT scans (please note details below).

Specify any mitigation strategies to minimize and/or manage risk(s): The exercise may result in muscle pulls or strains, or muscle soreness. Participants will be given a proper warm-up prior to exercising and qualified exercise trainers will supervise training sessions. Adequate rest will be given between training and testing sessions to ensure that participants' muscles are recovered by the next training session. Training will initially be quite light, and we will gradually increase the amount of training done per session over the first couple of weeks of training to allow participants' muscles to get used to the training and minimize muscle soreness. The effective radiation dose for DXA and pQCT scans at one measurement session is about 3.1 μSv (microSievert). Even in an unlikely situation, that would require all scans to be repeated (due to movement artifacts) at both measurement times, the total dose will be less than 7 μSv. We will scan each site a maximum of two times to minimize radiation exposure. The anticipated total effective dose is about 3.1 μSv and comparable to the amount of background radiation a person receives during a day from naturally-occurring sources in Saskatchewan. For reference, a cross-country flight could expose a person to about 30 μSv of radiation (http://www.hc-sc.gc.ca/hc-ps/ed-ud/respond/nuclea/measurements-mesures-eng.php). We are committed to practicing health and safety policies, procedures and guidelines that aim to reduce radiation to As Low as Reasonably Achievable (ALARA principle). Technician obtaining scans will stay a minimum of 1 meter away from the scanner while scanning is in the process to avoid scattered radiation exposure.

If applicable, justify the use of a placebo and/or washout: N/A

For double blind projects, identify provisions made to break the code in an emergency situation and indicate who holds the code:

Peer Review

The extent of peer review that is required will vary depending on the type of research being carried out. Typically, minimal risk research will not require peer review.

This project has received peer review:	☒ Yes ☐ No

If no, provide details: **Unavailable, SCPOR Leadership grant peer review comments were not shared**

If yes, submit the peer review along with the application.

PART 3: COMMUNITY ENGAGEMENT

Community Engagement

Is this research likely to affect the welfare of an Indigenous community, or communities, to which prospective participants belong? ☐ Yes ☒ No

If yes, researchers shall seek engagement with the relevant community as follows:

Outline the process to be followed for consulting with the appropriate community:

Describe how the community representatives will have the opportunity to participate in the interpretation of the data and the review of research findings before the completion of any reports or publications:

Describe how the final project results will be shared with the participating community(ies):

PART 4: RECRUITMENT

Participant Recruitment

Provide a detailed description of the method of recruitment. How/who will identify and contact prospective participants: * A research assistant (RA) will be in contact with sites to communicate with staff and residents interested in participating. Participants will be identified by independent living/retirement communities' staff and residents-representatives via advertisements by posters and emails. The residents interested in participating will contact RA via email or phone for the consent process.

Identify the anticipated number of participants to be enrolled at global site(s): * N/A

Identify the anticipated number of participants to be enrolled at local site(s): * 50

List the criteria for including participants: * Ambulatory individuals living in the independent living/retirement communities and able to safely perform exercise (pass the Get Active Questionnaire).

List the criteria for excluding potential participants: * Participants will be excluded if they are currently participating in pole walking or moderate-to-vigorous physical activity more than once per week. In addition, if the participant does not pass the Get Active Questionnaire, has heart conditions, angina during daily living, balance, and bone or joint problems, then a Physician Clearance Form is required to be filled out and signed by the participant's family physician. Those using assistive devices for mobility or diagnosed with Parkinson's disease will also be excluded.

PART 5: CONSENT

Consent Process

Specify who will explain the consent form and consent participants: Consent forms will be delivered to the participating sites by RA electronically or as hard copies. Site staff will share consent forms and individuals interested in to participate will have opportunity to ask questions or clarifications in the information session at site, or by contacting RA or PI (contact information provided in the consent forms and shared in the information sessions).

Explain where and under what circumstances consent will be obtained from participants: The RA will collect the signed copies of consent forms prior to the baseline measurements (the start of the pole walking intervention).

If a participant is unable to consent, explain the reason(s), describe the process by which their capacity will be assessed, identify who will consent on his/her behalf and describe the assent process: N/A

Describe any situation where the renewal of consent might be appropriate and how this would take place: N/A

How long will a participant have to decide whether or not to participate? If less than twenty-four hours, provide an explanation: As long as they want (prior to the pole walking intervention start)

Provide details on how participants can access, amend or withdraw their data and/or biological materials: Participants will receive feedback for their measures after the study measurements are completed. If participants wish to withdraw their data, they can contact the PI to communicate this withdrawal. PI can subsequently remove the data from any compiled data sheets, if this request is expressed prior to the publication of the results.

Provide the details of any compensation or reimbursements offered to the participants: No monetary compensation is provided.

Specify how and when participants will be able to obtain project results and whether the results will be individual and / or aggregate: * Participants will receive their own results via email. In addition, the article(s) of the study results will also be emailed to all participants. In addition to sharing the results via email, an information session will be held at the College of Kinesiology after completion of the intervention and data analysis in order to share the result of the study with participants and answer their questions.

PART 6: ALTERATION AND EXCEPTION TO CONSENT

Waiver of Consent

A waiver of participant consent is being requested: ☐ Yes ☒ No

If 'yes', justify the waiver by responding to the criteria below:

Access to identifiable information is essential to the research:

The use of identifiable information without the participants' consent is unlikely to adversely affect the welfare of individuals to whom the information relates:

The project team will take appropriate measures to protect the privacy of individuals and to safeguard the identifiable information:

The project team will comply with any known preferences previously expressed by individuals about any use of their information:

It is impossible or impracticable to seek consent from individuals to whom the information relates:

The researchers have obtained any other necessary permission for secondary use of information for research purposes:

Exception to Consent

An exception to the requirement to seek participant consent is being requested: ☐ Yes ☒ No

If 'yes', justify the exception by responding to the criteria below:

A serious threat to the prospective participant requires immediate intervention: ☐ Yes ☐ No

Either no standard efficacious care exists or the research offers a realistic possibility of direct benefit to the participant in comparison with standard care: ☐ Yes ☐ No

Either the risk is not greater than that involved in standard efficacious care, or it is clearly justified by the prospect for direct benefits to the participant: ☐ Yes ☐ No

The prospective participant is unconscious or lacks capacity to understand the risks, methods and purposes of the research project: ☐ Yes ☐ No

Third party authorization cannot be secured in sufficient time, despite diligent and documented efforts to do so: ☐ Yes ☐ No

No relevant prior directive by the participant is known to exist: ☐ Yes ☐ No

PART 7: BANKING OF BIOLOGICAL MATERIALS

Banking of Biological Materials

This project involves future undefined use of biological materials: ☐ Yes ☒ No

If 'yes', complete the following:

Describe the type, quantity of biological materials:

Describe how the biological materials will be collected, including the safety and invasiveness:

Describe the intended uses of the biological materials, including any commercial uses, and disclaimers:

Describe the measures employed to protect the privacy of and minimize risks to participants:

Specify the length of time the biological materials will be kept, how they will be preserved, location of storage (e.g., in Canada, outside Canada), and process for disposal:

Describe any anticipated linkage of biological materials with information about the participant:

Describe any plans for handling results and findings, including clinically relevant information and incidental findings:

If biological materials are culturally sensitive, describe what if any permissions are necessary:

If applicable, describe how the banked biological materials will be released to individuals external to the project:

If applicable, specify who has the custodianship of the biobank:

PART 8: DATA ACCESS, SECURITY AND STORAGE

Sources of Personal Health Information

Participant data collected prospectively:	☒ Yes ☐ No
Saskatchewan Health Authority:	☐ Yes ☒ No
Physician or other private health care professional office records:	☐ Yes ☒ No

Specify the Saskatchewan Health Authority facility(ies) and location(s):

Specify other source(s) of personal health information:

Data Access

List the research personnel responsible for abstracting project data and where the data abstraction will occur: Dr. Saija Kontulainen and RAs (including the student researcher) – College of Kinesiology

List the research personnel with access to identifiable project data: Dr. Saija Kontulainen and RAs

List the research personnel responsible for safeguarding the link to the source data: Dr. Saija Kontulainen and RAs

Security and Storage

Describe the data storage arrangements, while the project is ongoing: * Participants' email or mailing addresses will be requested in order to contact regarding ongoing and related future studies, inform participants of knowledge translation events or activities to be determined in collaboration with the community, and/or to send the results of this study upon completion. Contact information will be kept in a secured location separate from the data. Group emails will not be sent, nor mass mail outs, to protect the identity of the participants. Each participant will be assigned a code that will be kept separate from personal information and consent forms. The screening forms for individuals who are not eligible will be kept together in a separate folder. All of the screening forms and consent forms will be kept in a locked cabinet in the lab.

All digital data will be recorded on a secure password-protected, restricted access server drive on the University of Saskatchewan's internal server. Excel files of data will be encrypted, and password protected. Data will be kept on the server for a minimum of five years after the study is completed and data has been published. Only researchers approved for this study will be able to have access to the data. If participants do not want their data used for future studies, it will not affect their ability to participate in this study.

Specify how long data will be retained: Five (5) years from the date of publication of a report of the project research

If other, specify duration and provide justification:

The Principal Investigator will be responsible for the storage of data and/or biological materials:	☒ Yes ☐ No

If no, specify the reason and indicate who will be responsible:

Biological Materials and Data Transfer

Will data or biological materials be transferred outside the institution where they were collected: *	☐ Yes ☒ No

If 'yes', complete the following:

Organizations where data or biological materials will be transferred:

Indicate how data or biological materials will be transferred: Choose an item.

If 'Other' was selected, specify the method of transfer:

Mitigation Safeguards to Privacy Risks

Identify the safeguards/solutions to mitigate the risk to privacy.
Possible Safeguards/Solutions (check all that apply)
☒ Project personnel screening/agreements<br>☒ Access authorization procedures<br>☒ Designated systems administrator<br>☒ Passwords/screen timeouts<br>☒ System access audits/disclosure logs<br>☒ Secure mail/transport<br>☒ Firewall/virus protect<br>☒ Encrypted transmission<br>☐ Data collection tool and Master list stored in separate locations
☐ Aggregation levels<br>☐ Alternate identifiers
☐ Use of non-linkable elements or identifiers
☐ Confidentiality and security agreements for out-of-province recipients or storage providers

If applicable, describe any other mitigating strategies:

PART 9: DECLARATION OF PRINCIPAL INVESTIGATOR:

By submitting this application form, the Principal Investigator (PI) attests to the following:

- the PI accepts responsibility for the ethical conduct of this project and for the protection of the rights and welfare of the human participants who are directly or indirectly involved in this project.
- the PI will comply with all policies and guidelines of the University and affiliated institutions where this project will be conducted, as well as with all applicable federal and provincial laws regarding the protection of human participants in research.
- the PI will ensure that project personnel are qualified, appropriately trained and will adhere to the provisions of the Research Ethics Board-approved application.

- that adequate resources to protect participants (i.e., personnel, funding, time, equipment and space) are in place before implementing the research project, and that the research will stop if adequate resources become unavailable.
- any changes to the project, including the proposed method, consent process or recruitment procedures, will be reported to the Research Ethics Board for consideration in advance of implementation.
- will ensure that a status report will be submitted to the Research Ethics Board for consideration within one month of the current expiry date each year the project remains open, and upon project completion.
- if personal health information is requested, the PI assures that it is the minimum necessary to meet the research objective and will not be reused or disclosed to any parties other than those described in the Research Ethics Board-approved application, except as required by law.
- if a contract or grant related to this project is being reviewed by the University or Health Region, the PI understands a copy of the application, may be forwarded to the person responsible for the review of the contract or grant.

Date the form was completed: 2022-04-07
Name of Person who completed the form: Saija Kontulainen

If form submitted on behalf of the PI:
Mahdi Rostami Haji Abadi is authorized to prepare and submit this form on behalf of the Principal Investigator
Authorized person contact information:
Email: Phone:

DOCUMENT(S)

Provide a list of Documents that are being submitted along with this application:

1. Participant information and consent form
2. List of sites
3. Exercise guide
4. Consent process and inclusion/exclusion criteria checklist
5. Baseline testing checklist
6. Accelerometer wear time log
7. Accelerometer FAQ
8. RAND SF-36 questionnaire instrument
9. Exercise self-efficacy scale
10. Fall and Medication History Baseline
11. Fall and Medication History Follow-up
12. Falls efficacy measure
13. Feasibility/fidelity survey
14. Food frequency questionnaire
